# To the Members of the Medical Association of the State of Georgia

**Published:** 1868-02

**Authors:** W. F. Westmoreland

**Affiliations:** Chairman of Com.


### TO THE MEMBERS OF THE MEDICAL ASSOCIATION OF THE STATE OF GEORGIA.

At the last meeting of the Medical Association of the State of Georgia, a committee was appointed to revise the Constitution of the Association, of which the undersigned is Chairman. The committee has been unable to procure a copy of the Constitution, the Secretary not being able to furnish it, and an appeal is hereby made to the members of the Association, or any membea of the profession who may have the original, to forward it to the undersigned immediately, that the wishes of the Association may be fully met, at the next meeting.

W. F. WESTMORELAND,

Chairman of Com.

